# Consensus-based clustering and data aggregation in decentralized network of multi-agent systems

**DOI:** 10.7717/peerj-cs.1445

**Published:** 2023-08-28

**Authors:** Joshua Julian Damanik, Ming Chong Lim, Hyeon-Mun Jeong, Ho-Yeon Kim, Han-Lim Choi

**Affiliations:** Aerospace Engineering Department, Korea Advanced Institute of Science & Technology, Daejeon, South Korea

**Keywords:** Situational awareness, Clustering, Aggregation, Multi-agent systems, Optimization, Consensus, Distributed

## Abstract

Multi-agent systems are promising for applications in various fields. However, they require optimization algorithms that can handle large number of agents and heterogeneously connected networks in clustered environments. Planning algorithms performed in the decentralized communication model and clustered environment require precise knowledge about cluster information by compensating noise from other clusters. This article proposes a decentralized data aggregation algorithm using consensus method to perform COUNT and SUM aggregation in a clustered environment. The proposed algorithm introduces a trust value to perform accurate aggregation on cluster level. The correction parameter is used to adjust the accuracy of the solution and the computation time. The proposed algorithm is evaluated in simulations with large and sparse networks and small bandwidth. The results show that the proposed algorithm can achieve convergence on the aggregated data with reasonable accuracy and convergence time. In the future, the proposed tools will be useful for developing a robust decentralized task assignment algorithm in a heterogeneous multi-agent multi-task environment.

## Introduction

The field of multi-agent systems has gained increasing interest in recent years, particularly in the area of decentralized control (Xie & Liu, 2017). Although traditional centralized control systems are simple and robust (Oh, Park & Ahn, 2015), they have faced challenges in managing large numbers of agents and ensuring efficient performance (Ishaq et al., 2022). Thus, researchers have shifted their focus towards decentralized control methods which aim to distribute the computational and decision-making responsibilities among the agents in a local environment (Ponda et al., 2010). Applications of decentralized systems using multi-agent systems include air delivery systems (Oh et al., 2018; Damanik & Choi, 2021), search and rescue (Tomic et al., 2012), urban air mobility (Kim, Jeong & Choi, 2021), energy load management (Rasheed et al., 2019), surveillance (Samad, Bay & Godbole, 2007), and emergency relief systems (Bupe, Haddad & Rios-Gutierrez, 2015).

**Figure 1:**
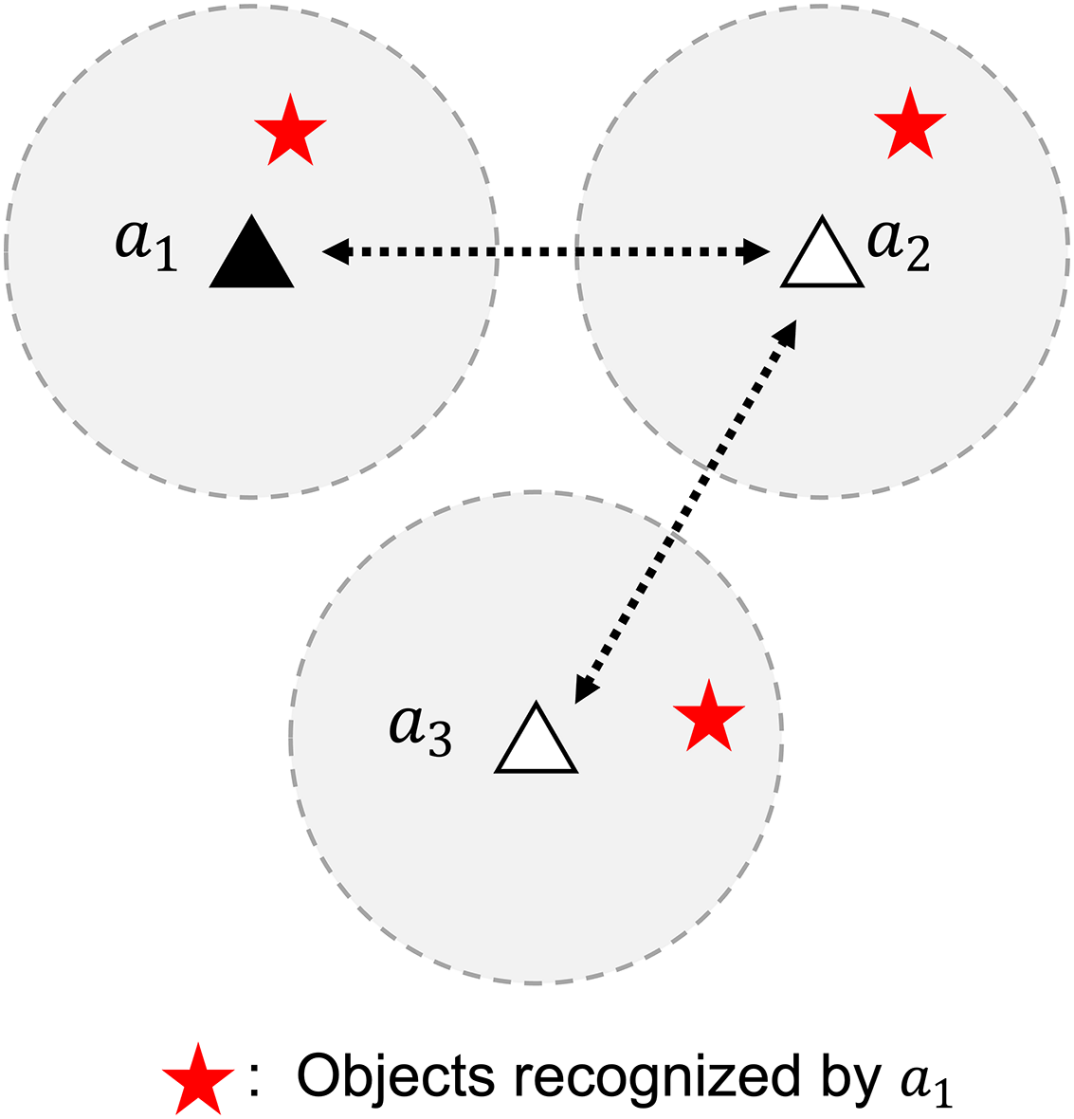
A problem of identifying objects in a decentralized network of multi-agent systems. Data aggregation enables agent 1 to recognize objects beyond its sensor range.

In addition to several advantages, decentralized control systems have one main challenge: communication between agents to perform coordination (Kwon & Hwang, 2020). Consensus (Choi, Brunet & How, 2009) may be one of the solutions to this. The sub-gradient method using consensus as proposed in Nedic & Ozdaglar (2009) is used to solve an optimization problem in a decentralized network of multi-agent systems. This algorithm implements the information evolution model proposed in Tsitsiklis (1984), which combines information from local neighbors using a weight rule. With this algorithm, the information from one agent is made available to every agent connected through communication links, either directly or indirectly, as illustrated in Fig. 1.

Based on the sub-gradient method, a decentralized clustering algorithm was proposed in Khawatmi, Zoubir & Sayed (2015), Khawatmi, Sayed & Zoubir (2017). This algorithm assumes that agents have no knowledge of the number of clusters and their respective information. Using a pairwise function, an agent determines the cluster in which it belongs and propagates its knowledge towards the consensus value of the cluster. This algorithm successfully performs clustering even in partially connected networks with few data exchanges and is comparable with centralized algorithms, including KMeans (MacQueen, 1967).

Decentralized local optimization, including decentralized multi-agent mission planning (Bertsekas, 1988; Choi, Brunet & How, 2009; Luo, Chakraborty & Sycara, 2014; Johnson, Choi & How, 2016) may require local knowledge to perform mission assignments in a decentralized network. However, highly complex tasks necessitating constraints, including coupled constraints as proposed in Whitten et al. (2011) and complex optimization objectives, including min-max tours as proposed in Prasad, Choi & Sundaram (2020) and Damanik & Choi (2021), require certain knowledge about the neighborhood which is difficult to acquire with conventional consensus in a partially connected network. To address this, data aggregation methods can be used.

The aim of data aggregation methods is to understand the data sensed by nodes and extract important information from it. Additionally, the system must be able to tackle various network restrictions. For example, in a partially connected network some data may be missing. The system must be capable of reconstructing the missing data using consensus between the agents. In a low energy network with limited bandwidth, data exchange must be executed efficiently to allow convergence to occur in a minimum number of inter-agent communications, without sacrificing the accuracy of the situational awareness. All of this must be possible without a substantial dependence on the central controller.

There are three types of data aggregation available: MIN/MAX aggregations, AVERAGE aggregations, and COUNT/SUM aggregations. MIN/MAX and AVERAGE aggregations are straightforward and various robust algorithms are available to execute these types of aggregation (Jesus, Baquero & Almeida, 2015; Fraser et al., 2012). Comparatively, COUNT/SUM aggregation is more challenging due to its sensitivity to duplicates, noise, and security attacks.

Several COUNT/SUM aggregation algorithms have been developed in order to solve the challenges posed, but each algorithm has its strengths and limitations. Token circulation, proposed in Dolev, Schiller & Welch (2006) and improved in Saha, Marshall & Reina (2019), is one approach for COUNT/SUM aggregation which implicates passing a token among the nodes and incrementing a counter every time the token is received. Probability density-based methods, such as probabilistic polling proposed in Friedman & Towsley (1999), Kalman-filter based polling in Alouf, Altman & Nain (2002), and maximum likelihood estimation in Baquero et al. (2012), require larger amounts of communication bandwidth to transmit probability density estimates between the nodes, limiting scalability.

Random walk-based algorithms, like Random-tour in Massoulié et al. (2006), Sample & Collide in Ganesh et al. (2007), and Capture-Recapture in Mane et al. (2005), have also been proposed for COUNT/SUM aggregation. These approaches involve nodes moving randomly through the network and incrementing a counter as they pass other nodes. However, these approaches are known to have low accuracy, particularly for larger numbers of nodes.

Recently, several gossip-based and consensus-based algorithms have been presented in order to overcome the limitations of previous methods. These approaches involve each node communicating with its neighbors, exchanging information and aggregating it over multiple rounds. Gossip-based algorithms such as Hop-sampling and interval density, proposed in Kostoulas et al. (2005) and Kostoulas et al. (2007) respectively, are examples of such algorithms. Consensus-based algorithms like Shames et al. (2012) and Lee et al. (2018) are other options. These algorithms do not require considerable bandwidth, produce accurate results, and converge in few iterations. However, no existing algorithm has been proposed for COUNT/SUM aggregation in a clustered network, which is common with large scale systems.

In order to tackle this limitation, this article presents a COUNT/SUM data aggregation in a clustered network of multi-agent systems. This algorithm takes into account the clustered network topology by using a notion of trust value, which reflects the trustworthiness of each node’s count value. The proposed algorithm requires relatively little communication and can achieve accurate results with few iterations, as proven through simulations and sensitivity analysis.

The rest of the article is organized as follows. ‘Proposed Algorithm’ proposes an algorithm that solves both clustering and data aggregation in a decentralized network. ‘Simulation’ details the simulation that was built and shows the simulation results. Finally, ‘Conclusion’ presents the concluding remarks.

## Proposed algorithm

In this section, we will explore the consensus-based data aggregation technique for multi-agent systems working in a clustered network environment. The goal of the algorithm is to perform COUNT or SUM calculations of a global information with only the available local information. By leveraging consensus on the aggregation variable and two supporting variables with neighboring agents, we can perform counting or summing of a variable from all connected members in a cluster. Additionally, the algorithm can perform an accurate data aggregation even in heterogeneous networks, wherein agents can be connected with members belonging to different clusters. Despite the noise, the algorithm can selectively filter the data and achieve convergence to the cluster aggregate value quickly.

The algorithm relies on the consensus of three important variables:

1. The (approximate cluster) centroid vector ( ${\omega _i}$). The centroid vector is the prediction on the centroid of an agent’s cluster. During the consensus, an agent compares the predicted centroid of both itself and its neighbor to find out if they are in the same cluster.
2. The aggregation contribution vector ( ${\phi _i}$). The aggregation contribution variable is a measure of each agent’s contribution to the aggregation dynamics of the cluster, and it plays a critical role in determining the overall behavior of the system by ensuring that each agent’s contribution is properly accounted for.
3. The aggregate value ( ${\psi _i}$). On the other hand, the aggregate variable is the COUNT or SUM of the data being aggregated. This variable symbolizes the final result of the aggregation process, providing a comprehensive summary of the data collected and processed by the agents.

The multi-agent system is modeled as a graph $G = (V,E)$, where *V* is the set of vertices or nodes representing agents, and *E* is the set of edges that signify the interactions between the agents. Each edge $e \in E$ is a tuple $(u,v)$, where $u$ and $v$ are nodes in *V*, indicating that agents represented by $u$ and $v$ are interacting. The graph can be weighted or unweighted, relying on whether or not the edges have a specific value or weight associated with them, which can represent the strength or significance of the interaction between the agents.

**Figure 2:**
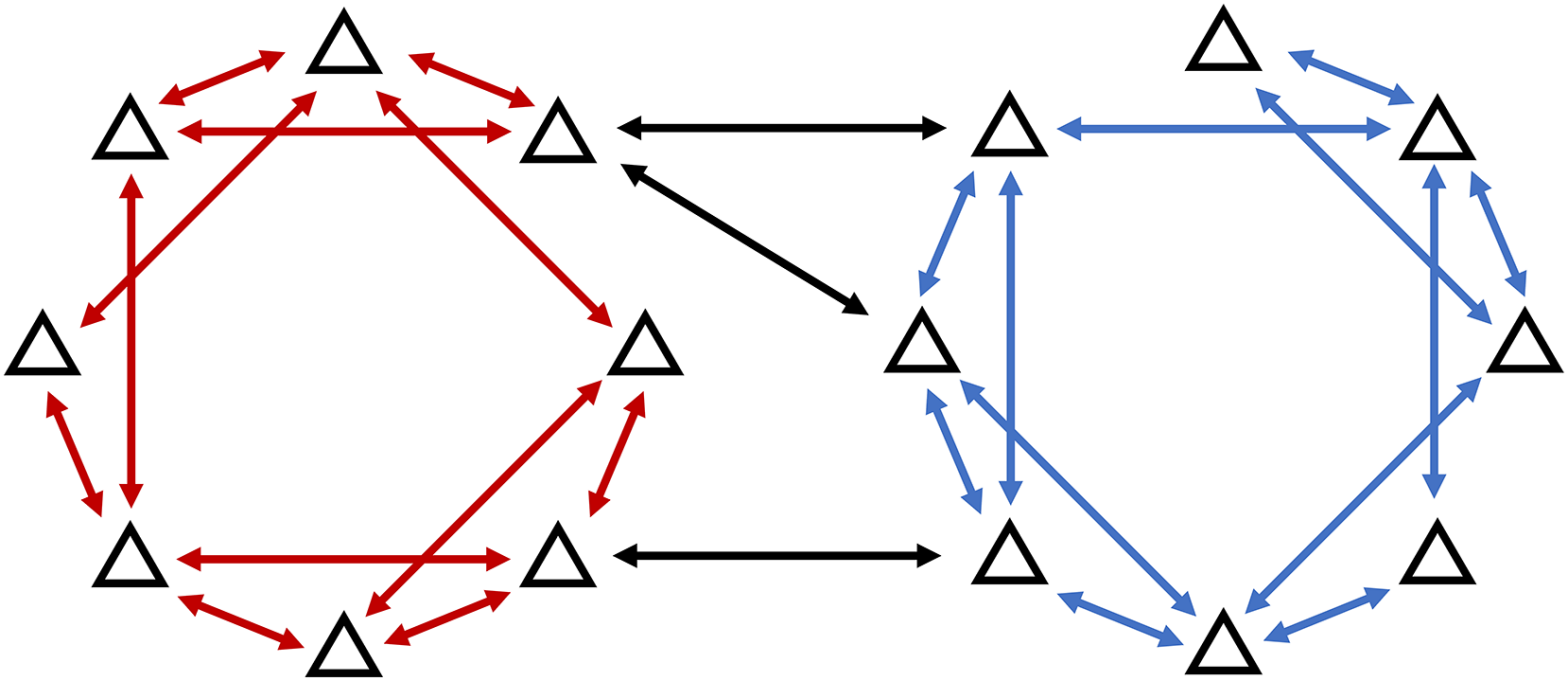
Heterogeneous network model allows for the communication between agents in different clusters.

Each node $i \in V$ in the environment belongs to a cluster ${c_i}$ from the cluster set $C = \{ 1, \ldots ,m\}$ in one-to-one relationships. Each cluster $c$ is identified with its centroid ${w_c} \in {R^k}$, with $k$ being the number of centroid’s dimensions. In this article, we assume that the network is heterogeneous, wherein there are edges between agents in different clusters. Let ${N_i}$ denote the set of $i$’s adjacent nodes, wherein node $j \in {N_i}$ can belong to any cluster, it does not have to be in the same cluster with $i$ (Fig. 2).

**Figure 3:**
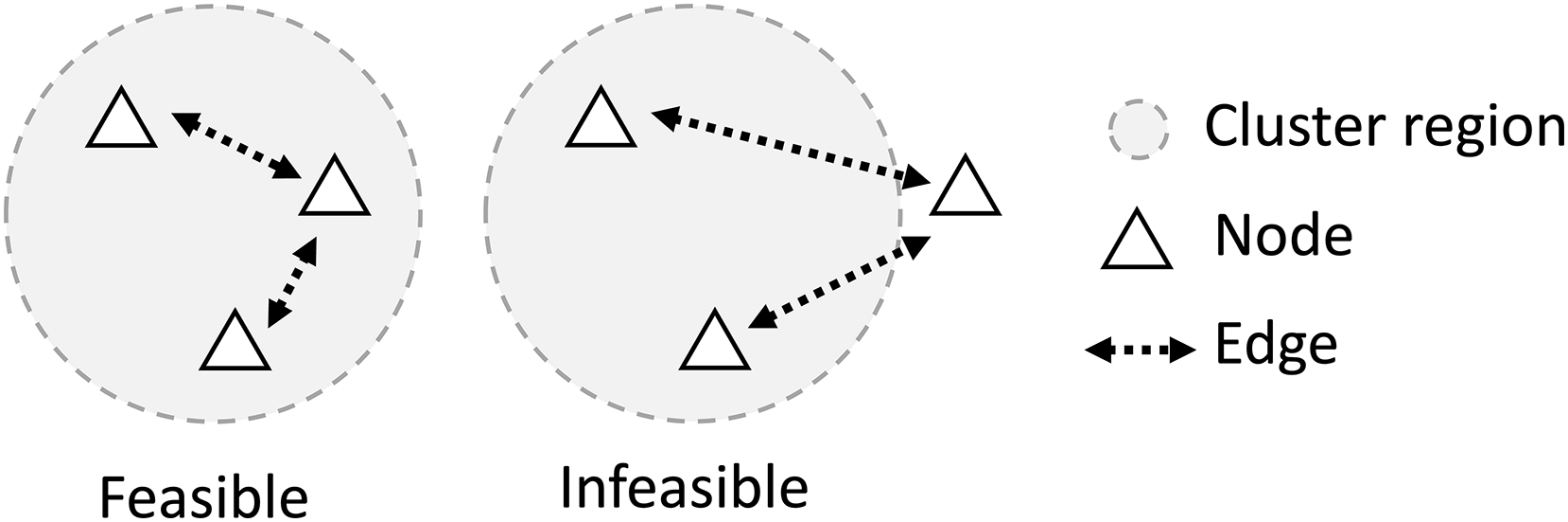
For a feasible network, all nodes in the cluster must be connected with at least one member of the same cluster, making the group of agents on the right unfeasible.

It is also assumed that there must be a set of edges that can connect every pair of agents in the cluster. This implies that communication between two agents in the same cluster is always possible, either directly or through several hops between agents in the cluster as illustrated in Fig. 3.

In cooperative multi-agent systems, it is assumed that the system minimizes the total cost of the entire system, which is the sum of each agent’s cost. Specifically, this optimization problem can be expressed as ${\min _{i \in V}}{f_i}(x)$ where ${f_i}(x)$ is the individual cost function of each agent.

In a decentralized network, the global knowledge of the environment’s state $x$ is not always available, thus agents must generate an estimate of the environment status, denoted as ${x_i}$. Assuming that the agent updates its estimate in a discrete time domain, every time step an agent updates its estimate using the relation

(1) $${x_i}(t + 1) = \sum\limits_{j \in V} {{a_{ij}}} {x_j}(t) - \alpha \nabla {f_i}({x_i}(t))$$

where ${a_i} = [{a_{i1}}, \ldots ,{a_{in}}]$ is a weight vector used to calculate the average estimates from agent $i$’s neighbors. The weight vector must comply with the weight rules as follows

(2) $$\sum\limits_{j \in V} {{a_{ij}}} = 1$$

(3) $${a_{ij}} = \left\{ \matrix{
  {\eta _j},\,\,\,\,\,\,{\mathrm{if}}\;j = i\;{\mathrm{or}}\;j\;{\mathrm{is}}\;{\mathrm{agent}}\;i{\mathrm{'s}}\;{\mathrm{neighbor}} \hfill \cr 
  {\mathrm{0}},\,\,\,\,\,\,\,\,{\mathrm{otherwise}} \hfill \cr}  \right.$$

where ${\eta _j}$ is any scalar between 0 and 1 ( $0\lt{\eta _j}\lt1,\forall j \in V$).

### Consensus on centroid vector

In decentralized systems, agents do not know the exact values of cluster centroids and members of the clusters. Instead, they conduct consensus on an estimated centroid value and compare it with their neighbors to establish whether they belong to the same cluster. Let ${x_i} \in {R^k}$ be agent $i$’s state used for the basis of clustering, and ${\omega _i} \in {R^k}$ be the predicted centroid vector of its cluster ${c_i}$. Then, we can define a variable called trust value, denoted as ${v_{ij}}$, that compares the predicted values of clusters of agents $i$ and $j$. The trust value must satisfy the following equation.

(4) $${v_{ij}}(t) = 1 \Rightarrow ||{\omega _i}(t) - {\omega _j}(t)|| = 0$$

The trust function can be employed to identify whether an agent $j$ is in the same cluster as agent $i$ or not. We can define a scalar $\varepsilon$ as a boundary. Agents $i$ and $j$ are said to be within the same cluster if ${v_{ij}} \gt \varepsilon$. We can utilize any convex function to define the trust function as long as it satisfies Eq. (4).

(5) $${v_{ij}}(t) = g({\omega _i}(t),{\omega _j}(t),t)$$

At the initial stage, each agent sets the centroid vector to its state ${\omega _i}(0) = {x_i}$. For each time iteration $t = 1,2, \ldots$, every agent performs consensus on the centroid vector ${\omega _i}$ with their neighbors ${N_i}$ and updates the vector iteratively using the following equation.

(6) $${\omega _i}(t + 1) = {1 \over {\sum\nolimits_{j \in {N_i} \cup i} {{v_{ij}}} }}\sum\limits_{j \in {N_i} \cup i} {{v_{ij}}} (t){\omega _j}\quad \forall i \in V$$

### Consensus on aggregation contribution vector

The data aggregation model proposed in this article is based on the network counting system proposed in Lee et al. (2018), using blended dynamics (Eq. (7)):

(7) $$\dot s = - {s \over n} + 1$$

Using the steady-state theorem, it is easy to prove that $\mathop {\lim }\nolimits_{t \to \infty } s(t) = n$. Developing the consensus model based on this dynamics leads to convergence to the number of members in a cluster. In this article, we extended this model in three ways:

1. In the original article, the counting mechanism requires a leader to generate dynamic feedback in order to reach convergence. In this article, we allow every member of the cluster to contribute a fraction of the dynamic feedback, thus removing the need for a cluster leader.
2. We extended the model to allow summing by introducing a state constant $y \in R$ on the right-hand side of the dynamics in Eq. (7).
3. The original model assumes a homogeneous network, where no cluster notation is defined. In this article, we extended the blended model to work with heterogeneous networks with multiple clusters by implementing trust values into the consensus models.

First, to determine the fraction of contribution of each node on the blended dynamics, we introduce a contribution vector ${\phi _i} = [{\phi _{i1}}, \ldots ,{\phi _{in}}]$ that satisfies

(8) $$\sum\limits_{j \in V} {{\phi _{ij}}} = 1,\quad \forall i \in V$$

The vector ${\phi _i}$ is initially decided by each agent $i$ using Eq. (9) and then updated using the consensus rule based on the structure of the network. The idea is to generate a trust value that is proportional to the degree of the node. At the initial stage, each agent decides a vector such that

(9) $${\phi _{ij}} = \left\{ \matrix{
  1,\,\,\,\,\,{\mathrm{for}}\;{\mathrm{j = i}} \hfill \cr 
  0,{\mkern 1mu} {\mkern 1mu} \,\,\,\,{\mathrm{otherwise}} \hfill \cr}  \right.$$

And then, at every time step, agents perform consensus with their neighbors using Eq. (10) below.

(10) $${\phi _i}(t + 1) = {1 \over {\sum\nolimits_{j \in {N_i} \cup i} {{v_{ij}}} (t)}}\sum\limits_{j \in {N_i} \cup i} {{v_{ij}}} (t){\phi _j}(t),\quad \forall i \in V$$

The consensus of the contribution vector might require a slightly higher bandwidth, as an entire vector ${\phi _i}$ must be exchanged between agents and their neighbors. However, by ensuring that the centroid vectors are converged, where the prediction distance between agents in a cluster is very small, the consensus on contribution vectors can reach convergence very quickly.

### Consensus on the aggregation value

The consensus on the aggregation value is a critical aspect in many decentralized systems. The objective is to collect and sum the state variables of all agents in a cluster and arrive at a single value. The state variable of each agent to be aggregated is represented by ${y_i}$, and the sum of all agents’ states in a cluster $c \in C$ is represented by ${Y_c}$, as defined in Eq. (11):

(11) $${Y_c} = \sum\limits_{i \in c} {{y_i}} ,\quad \forall c \in C$$

Given ${\phi _i}$ as the contribution vector known by agent $i$, the agent decides its ratio of contribution feedback to the aggregation dynamics (Eq. (7)) as ${\phi _{ii}}$. At every time step $t = 1,2 \ldots$, the agents generate an addition to the consensus value.

To determine the contribution of each agent to the aggregation process, a contribution vector ${\phi _i}$ is used. This vector represents the ratio of contribution feedback from each agent to the aggregation dynamics. At every time step, each agent generates an addition to the consensus value by taking the ratio value of the agent $i$ as ${\phi _{ii}}$.

Each agent calculates the prediction value of ${Y_c}$ as ${\psi _i}$, which represents the approximate value of the sum of ${y_j}$ for all agents in the cluster ${C_i}$. During the iteration process, agents exchange their prediction data using Eq. (12). This equation ensures that each agent has an updated and accurate prediction value of ${Y_c}$.

(12) $$\psi_{i}(t+1)=\frac{1}{\sum_{{j\in N_{i}\cup i}{v_{ij}(t)}} }{\sum_{j\in N_{i}\cup i}}v_{ij}(t)\psi_{j}(t)+\epsilon(y_{i}-\phi_{ii}(t)),\quad\forall i\in V$$

Equation (12) represents the aggregation of the prediction value of each agent during the iteration process. Here, ${\psi _i}(t + 1)$ is the prediction value of the sum of all agents’ states ${y_i}$ in the cluster $c \in C$ at the $(t + 1)$-th time step. The equation computes the new prediction value based on the previous prediction values of the neighboring agents and the local state of the agent.

The term ${1 \over {\sum\nolimits_{j \in {N_i} \cup i} {{v_{ij}}} (t)}}\sum\nolimits_{j \in {N_i} \cup i} {{v_{ij}}} (t){\psi _j}(t)$ represents a weighted average of the prediction values of the neighboring agents and the current agent. The weight ${v_{ij}}(t)$ determines the influence of the prediction value of each neighboring agent on the new prediction value of the current agent.

The term $\varepsilon(y_{i}-\phi_{ii}(t)\psi_{i}(t))$ represents the correction term that the agent applies to its previous prediction value based on its own local state ${y_i}$. ${\phi _{ii}}(t)$ is the ratio of contribution feedback to the aggregation dynamics, and $\varepsilon$ is a small positive constant that determines the magnitude of the correction term.

Equation (12) is calculated for every agent $i \in V$, where *V* is the set of all agents in the network. The iteration process continues until the prediction values of all agents converge to the same value, which represents the consensus on the sum of all agents’ states ${y_i}$.

The complete algorithm is shown in Algorithm 1.

**Algorithm 1:** Decentralized data aggregation in clustered network for each agent *i*

**Require: ** $X_{i}, \,Y_{i},\, \varepsilon$
1: ${\omega _i}(0) \leftarrow {X_i}$
2: $${\phi _{ij}}(0) \leftarrow \left\{ \matrix{ 1,\,\,\,\,{\mathrm{if}}\;{\mathrm{j = i}} \hfill \cr 0,\,\,\,{\mathrm{otherwise}} \hfill \cr} \right.$$
3: ${\psi _i}(0) \leftarrow {Y_i}$
4: $t \leftarrow 0$
5: **while** ${\psi _i}$ is not converged **do**
6: ${\hat \omega _i}(t) \leftarrow {\omega _i}(t)$
7: ${\hat \phi _i}(t) \leftarrow {\phi _i}(t)$
8: ${\hat \psi _i}(t)\leftarrow \psi_{i}(t)+\varepsilon(Y_{i}-\phi_{ii}(t)\psi_{i}(t))$
9: ${\hat v_i} \leftarrow 1$
10: **for** $j \in {N_i}$ **do**
**Receive** ${\omega _j}(t)$ **from** *j*
11: ${v_{ij}}(t) = f\left( {{\omega _i}(t),{\omega _j}(t),t} \right)$
12: ${\hat v_i} \leftarrow {\hat v_i} + {v_{ij}}$
13: **if** ${\phi _i}$ is not converged **then**
14: **Receive** ${\phi _j}(t)$ **from** *j*
15: ${\hat \omega _i}(t) \leftarrow {\hat \omega _i}(t) + {v_{ij}}{\omega _j}(t)$
16: ${\hat \phi _i}(t) \leftarrow {\hat \phi _i}(t) + {v_{ij}}{\phi _j}(t)$
17: **end if**
18: **Receive** ${\psi _j}(t)$ **from** *j*
19: ${\hat \psi _i}(t) \leftarrow {\hat \psi _i}(t) + {v_{ij}}{\psi _j}(t)$
20: **end for**
21: **if** ${\phi _i}$ is not converged **then**
22: ${\omega _i}(t + 1) \leftarrow {\hat \omega _i}(t)/{\hat v_i}$
23: ${\phi _i}(t + 1) \leftarrow {\hat \phi _i}(t)/{\hat v_i}$
24: **else**
25: ${\omega _i}(t + 1) \leftarrow {\hat \omega _i}(t)$
26: ${\phi _i}(t + 1) \leftarrow {\hat \phi _i}(t)$
27: **end if**
28: ${\psi _i}(t + 1) \leftarrow {\hat \psi _i}(t)/{\hat v_i}$
29: $t \leftarrow t + 1$
30: **end while**

## Simulation

The simulation environment was designed to emulate the proposed algorithm in a clustered network of multi-agent systems and to test the algorithms under various conditions. The results obtained from the simulations provide valuable insights into the efficiency and scalability of the algorithms and help to validate the theoretical findings presented in the article. This section will provide a detailed description of the simulation setup, the conditions tested, and the results obtained.

The simulation first generates sample data of 100 agents with locations in 2D domains using the make_blobs function from the sklearn.datasets module. A network with 100 agents is considered large and the algorithm can solve the data aggregation in a reasonable number of iterations. The sample data is sorted based on its cluster labels and stored in a state matrix *X* of size $100 \times 2$ and a cluster label vector $c$ of size $100 \times 1$.

**Figure 4:**
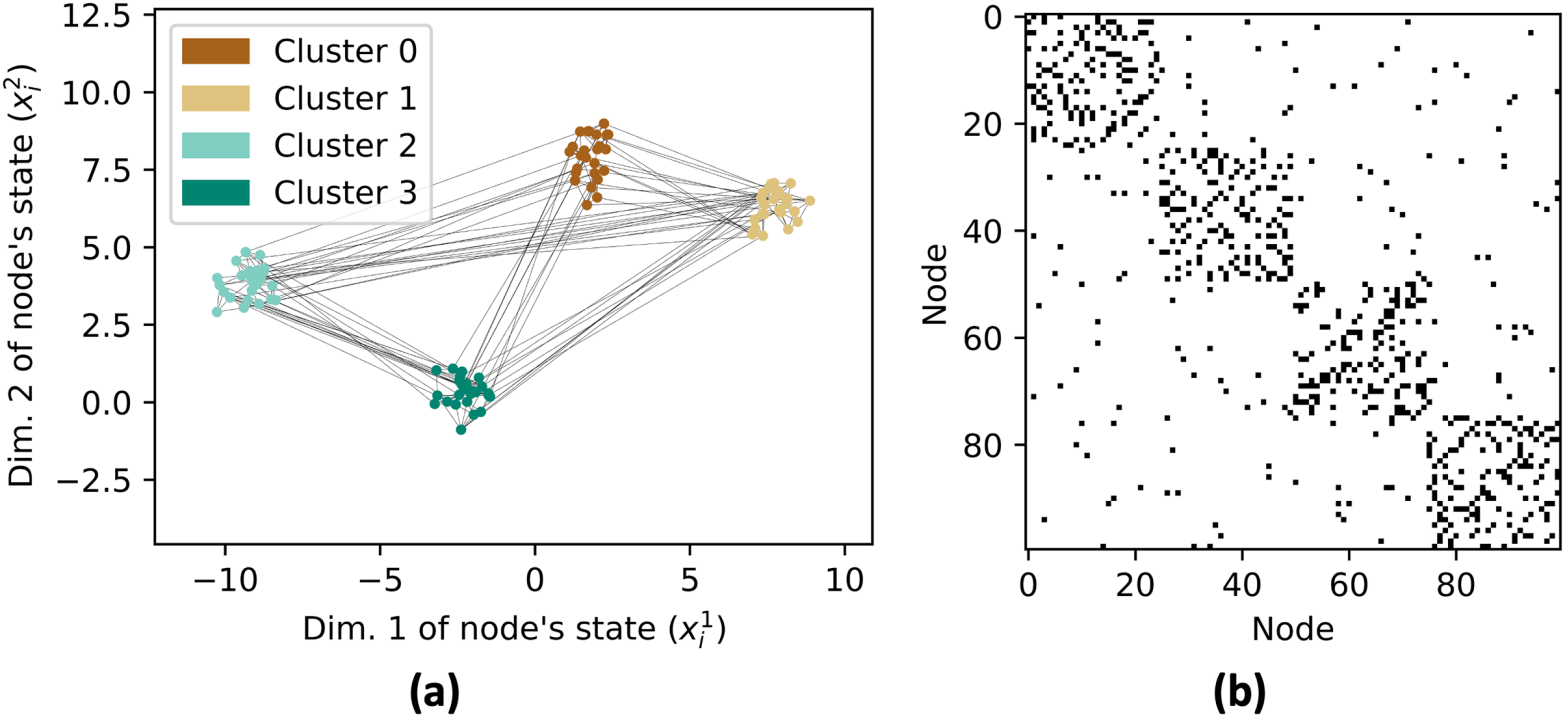
Network graph *G* consisting of 4 clusters with 25 nodes each. Each node has edges with its nearest 10 neighbors and 10% probability with any random nodes as shown in (A). The resulting adjacency matrix is shown in (B).

Next, a graph *G* is created with 100 nodes with *X* as the location of nodes (Fig. 4A). The edges in the graph are determined based on the distances between nodes. To generate strong connections within a cluster, the NearestNeighbors class from the sklearn.neighbors module is used to find the nearest neighbors for each node and we connect each agent with their 10 nearest neighbors. The graph is also augmented with random edges with a probability of 0.1% to increase its inter-cluster connectivity. The adjacency matrix of the graph is shown in Fig. 4B.

**Figure 5:**
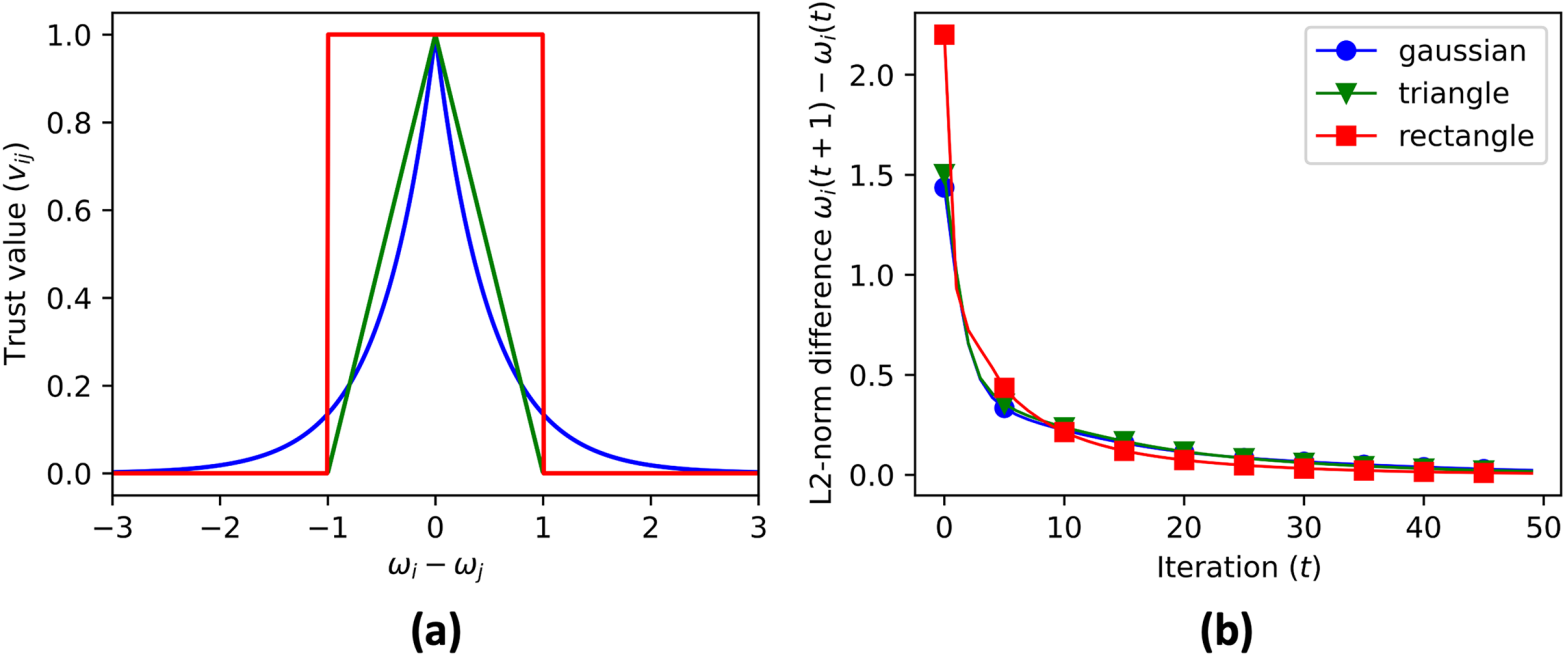
(A) The three convex functions used to generate trust value: gaussian (Eq. (13)), triangle (Eq. (14)), and rectangle (Eq. (14)) functions. The convergence rate of cluster centroid prediction is shown in (B).

The code then initializes the cluster centroid prediction $\omega$, contribution matrix $\phi$, and aggregate matrix $\psi$. These matrices are updated in each iteration of the data aggregation process using Eqs. (6), (10), and (12). The data to be aggregated is stored in a vector $y \in {R^n}$ with initial value equal to ID of each node, ${y_i} = i,\;\forall i$. The real aggregate of the data is calculated and stored in a vector ${Y_i} = \sum\nolimits_{i \in {C_i}} {{y_i}} ,\;\forall i$ for error reference. The trust value ${v_{ij}}$ is calculated using three different convex functions (Fig. 5A): Gaussian (Eq. (13)), triangular (Eq. (14)), and rectangular (Eq. (15)).

1. Gaussian function

(13) $${v_{ij}} = \exp \left( { - {{\Vert{\omega _i} - {\omega _j}\Vert{_2}} \over {2{\sigma ^2}}}} \right)$$

2. Triangular function

(14) $${V_{ij}} = \left\{ \matrix{
  1 - {{{{\left\| {{\omega _i} - {\omega _j}} \right\|}_2}} \over {2\sigma }},\,\,\,\,\,{\mathrm{if}}\;{\left\| {{\omega _{\mathrm{i}}}{\mathrm{ - }}{\omega _{\mathrm{j}}}} \right\|_{\mathrm{2}}} \le {\mathrm{2}}\sigma  \hfill \cr 
  0,\,\,\,\,\,\,\,\,\,\,\,\,\,\,\,\,\,\,\,\,\,\,\,\,\,\,\,\,\,\,\,{\mathrm{otherwise}} \hfill \cr}  \right.$$

3. Rectangular function

(15) $${v_{ij}} = \left\{ \matrix{
  1,\,\,\,\,\,\,{\mathrm{if}}\;{\left\| {{\omega _{\mathrm{i}}}{\mathrm{ - }}{\omega _{\mathrm{j}}}} \right\|_{\mathrm{2}}} \le {\mathrm{2}}\sigma  \hfill \cr 
  0,\,\,\,\,\,{\mathrm{otherwise}} \hfill \cr}  \right.$$

**Figure 6:**
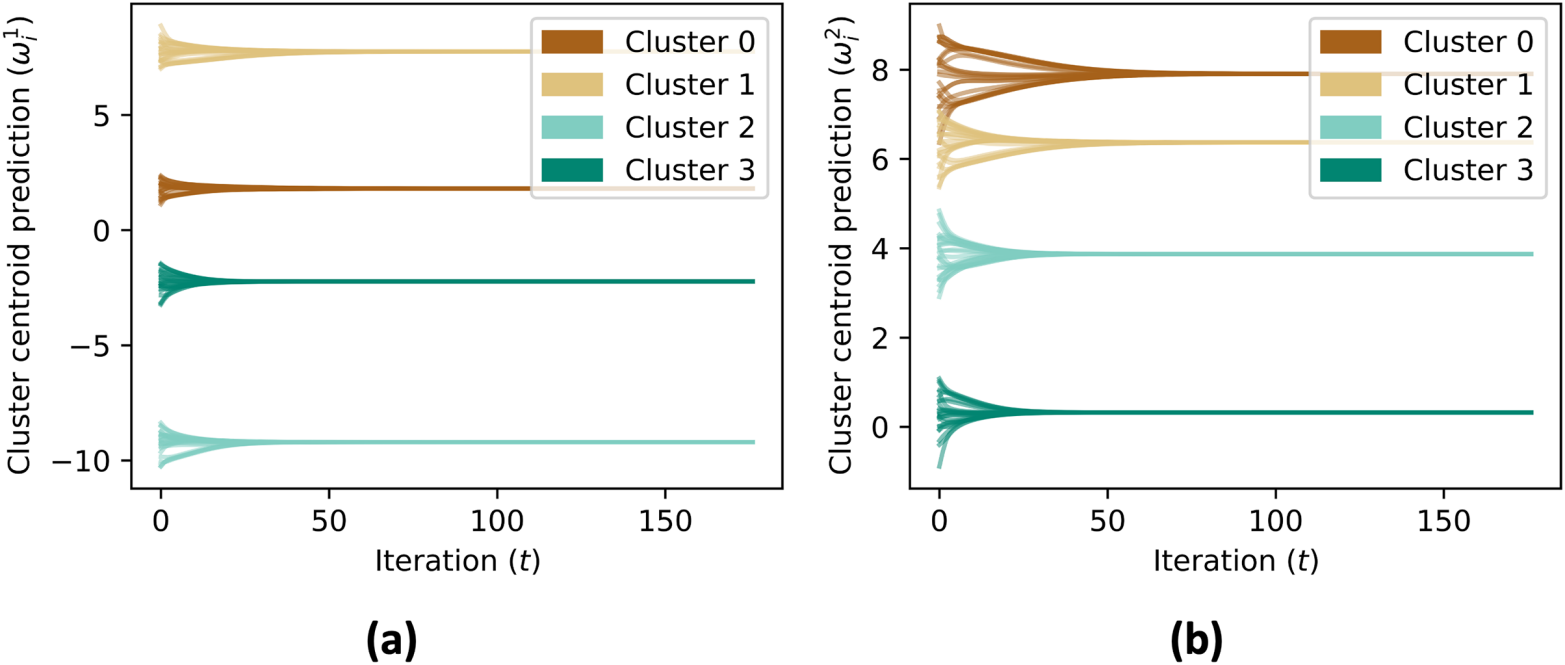
(A and B) The prediction of cluster centroid by all nodes. In initial, the centroid is defined to each node’s state ( ${{\omega _i} = {x_i}}$). Using consensus rule on Eq. (6), the centroid prediction converged to mean value of each member’s state of the cluster.

**Figure 7:**
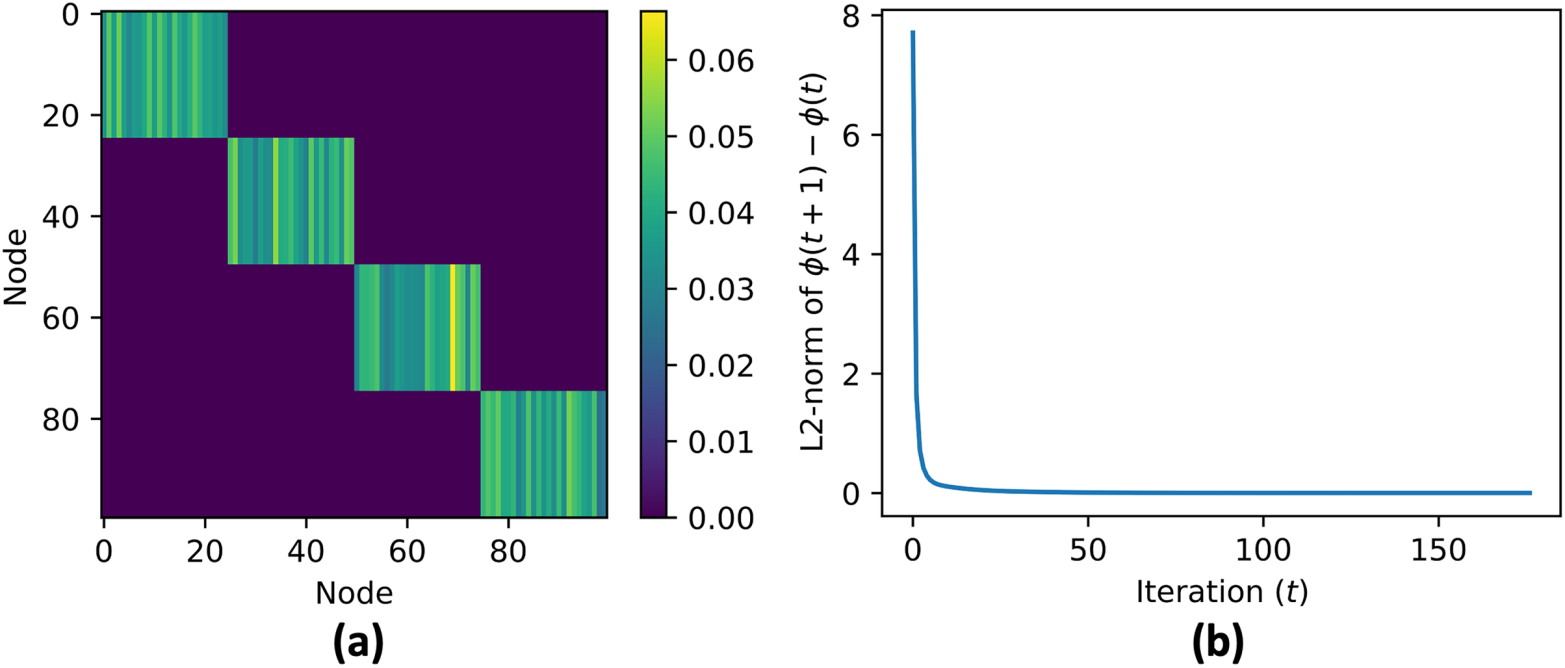
(A and B) Contribution vector value at convergence ${\phi (\infty )}$. For every agents in the same cluster the contribution vector converge to the same value.

The data aggregation process is repeated for a maximum number of iterations, specified by MAX_ITER. The code checks if the contribution vectors has converged by comparing the updated values with the previous values, and if the difference is within a given tolerance, the process terminates. After the convergence, the simulation skips the consensus on the contribution vector, and continues the consensus for the aggregate value until convergence. Figure 6 shows the cluster centroid predictions of all agents, over the course of the iterations. Figure 5B shows the convergence rate of the centroid cluster prediction using three different trust functions, and Fig. 7 shows the contribution vector of all agents at convergence.

**Figure 8:**
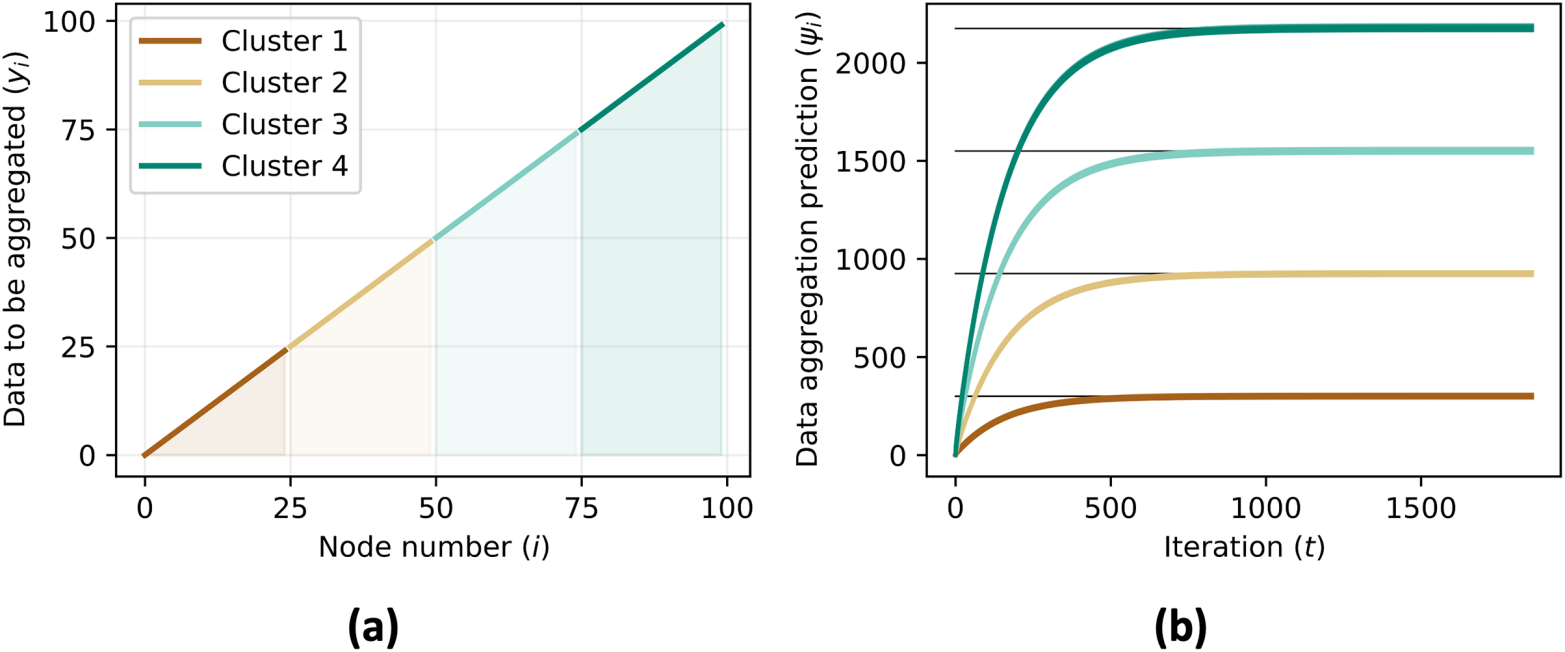
Data to be aggregated (A) for each nodes equals to the ID of each agents ( ${{y_i} = i,\;\forall i}$) and the data aggregation (B) converges to the sum of data to be aggregated of all agents in the same cluster, ${{\psi _i} \, {\simeq \sum\nolimits_{j \in {C_i}} {y_j}}}$.

The simulation also calculates the mean squared error between the real aggregate value and the predicted aggregate value, which is a measure of the difference between the two. Figure 8B shows the mean squared error for each iteration.

We also provide sensitivity analysis of our proposed algorithm. Sensitivity analysis is a crucial step in evaluating the performance of the proposed algorithms for decentralized data aggregation in clustered networks of multi-agent systems. In this study, sensitivity analysis was performed to investigate the effect of various parameters on the accuracy of data aggregation. The parameters considered in the sensitivity analysis include the value of epsilon (a threshold value used in the algorithm), the number of connected neighbors, the probability of random edge generation, and the number of sample nodes.

**Figure 9:**
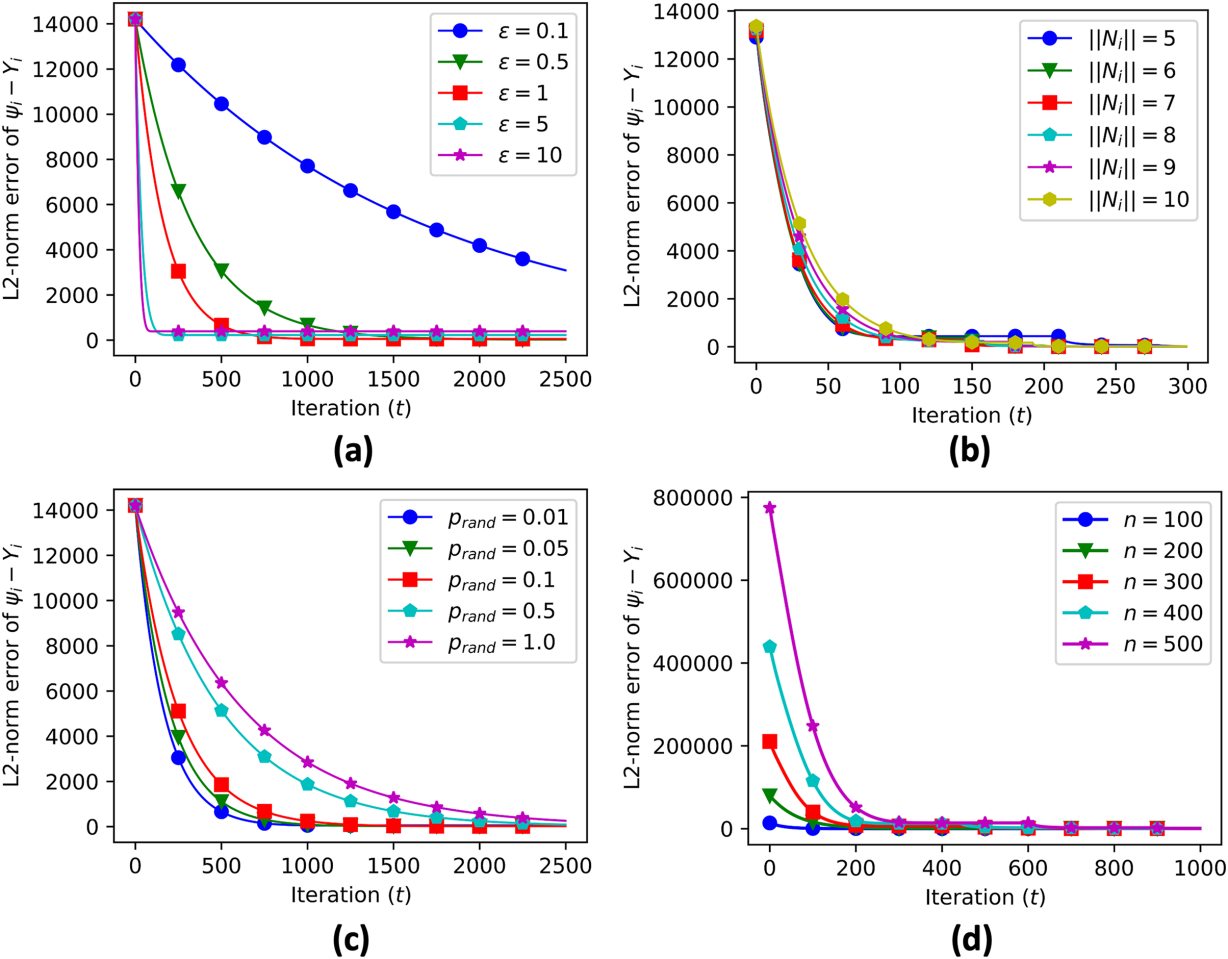
Sensitivity analysis of error propagation with various parameters, including data aggregation step size ${\varepsilon}$ (A), number of connected neighbors (B), probability of random edge generation (C), and number of sample nodes (D).

A sensitivity analysis was conducted to evaluate the performance of the proposed algorithm. The parameters considered in the analysis were: $\varepsilon$ (data aggregation step), the number of connected neighbors ( $||{N_i}||$), the number of sample nodes ( $n$), and the probability of random edge generation ( ${p_{rand}}$). The results of the sensitivity analysis showed that the data aggregation error was sensitive to all of these parameters, as shown in Table 1. In particular, as the value of $\varepsilon$ increased, the convergence time reduced significantly while increasing the convergence error (Fig. 9A). The sensitivity analysis also showed that various numbers of connected neighbors (Fig. 9B), probabilities of random edge generation (Fig. 9C), and number of sample nodes (Fig. 9D) still led to similar convergence values, demonstrating the robustness of the algorithm in heterogeneous networks.

**Table 1:** Sensitivity analysis is performed to measure the convergence time of contribution vector ( ${\phi}$) and data aggregation value ( ${\psi}$) with various value of data aggregation step size ${\varepsilon}$, number of neighbors ( ${\Vert{N_i}\Vert}$), number of nodes ( ${n}$), and probability of random edge generation ( ${{p_{rand}}}$).

${\varepsilon}$	$ {\Vert{N_i}\Vert}$	$ {n}$	${p_{rand}}$	Convergence time		L2-Norm error ${\Vert\psi - Y\Vert{_2}}$
				${\phi}$ (iteration)	${\psi}$ (iteration)	
0.1	5	100	0.01	159	26,236	5.325308
0.5	5	100	0.01	159	5,769	26.164664
1	5	100	0.01	159	2,994	51.233616
5	5	100	0.01	159	648	222.297639
10	5	100	0.01	159	343	389.116116
1	5	100	0.01	299	299	15.976597
1	6	100	0.01	299	299	3.558554
1	7	100	0.01	299	299	9.557468
1	8	100	0.01	299	299	11.039423
1	9	100	0.01	299	299	13.091504
1	10	100	0.01	299	299	16.758241
1	5	100	0.01	299	299	38.259445
1	5	200	0.01	299	299	729.815696
1	5	300	0.01	999	999	30.42748
1	5	400	0.01	999	999	236.236123
1	5	500	0.01	999	999	282.063527
1	5	100	0.01	159	2,994	51.233616
1	5	100	0.05	57	3,585	28.155643
1	5	100	0.1	31	4,447	19.498971
1	5	100	0.5	11	8,529	9.144341
1	5	100	1	6	9,999	6.898491

## Concluding remarks

This article proposed an algorithm to perform data clustering and aggregation in a decentralized network of multi-agent systems, using consensus methods on three key variables: approximate cluster centroid vectors, aggregation contribution vectors, and aggregate values. The trust value used in the consensus rules enables data aggregation in a heterogeneously connected clustered network. Thus, data aggregation is performed in the cluster scale while still allowing inter-cluster communication.

The accuracy and convergence rate of the algorithm are dependent on the data aggregation step size constant $\varepsilon$. A bigger value of $\varepsilon$ results in faster convergence time, but with higher aggregation errors, and vice versa. The main advantages of the proposed algorithm include the fact that it does not require many data transfers between its neighbors and that the data aggregation can reach convergence in a reasonable number of iterations, even in heterogeneous networks. These advantages allow both clustering and data aggregation in a decentralized network with limited bandwidth.

Future work includes extending the proposed algorithm to work in switching networks and improving the accuracy and convergence time by modifying the consensus rules for data aggregation. Furthermore, there are various applications which will benefit from this algorithm, such as decentralized task allocation algorithms and coordination algorithms in multi-agent systems.

## Additional Information and Declarations

### Data Availability

The following information was supplied regarding data availability: The data is available at GitHub and Zenodo: https://github.com/joshuadamanik/peerj.git. Joshua J. Damanik. (2023). joshuadamanik/peerj: release (release). Zenodo. https://doi.org/10.5281/zenodo.7765749.
